# Influence of lymphadenectomy on survival and recurrence in patients with early-stage epithelial ovarian cancer: a meta-analysis

**DOI:** 10.1186/s12905-023-02615-6

**Published:** 2023-09-04

**Authors:** Chenchen Yang, Ting Zhang, Aifeng Gong, Can Shi

**Affiliations:** 1 Department of Emergency, The Affiliated Huai’an No. 1 People’s Hospital of Nanjing Medical University, Huai’an, 223300 Jiangsu China; 2 Department of Obstetrics and Gynecology, The Affiliated Huai’an No. 1 People’s Hospital of Nanjing Medical University, Huanghe Road West,, Huai’an, 223300 Jiangsu China; 3 Department of General Practice, The Affiliated Huai’an No. 1 People’s Hospital of Nanjing Medical University, Huai’an, 223300 Jiangsu China

**Keywords:** Early-stage epithelial ovarian cancer, Lymphadenectomy, Overall survival, Progression-free survival, Recurrence rate

## Abstract

**Background:**

This meta-analysis aimed to evaluate the effectiveness of lymphadenectomy on survival and recurrence in patients with early-stage epithelial ovarian cancer (eEOC).

**Methods:**

Relevant studies were searched from four online databases. Hazard ratios (HRs) with 95% confidence intervals (CIs) or risk ratios (RRs) with 95% CIs were used to evaluate the effects of lymphadenectomy on overall survival (OS), progression-free survival (PFS), and recurrence rates. A subgroup analysis was performed to explore the sources of heterogeneity, followed by sensitivity and publication bias assessments.

**Results:**

Fourteen articles involving 22,178 subjects were included. Meta-analysis revealed that lymphadenectomy was significantly associated with improved OS (HR = 0.72; 95% CI:0.61, 0.84; *P* < 0.001), improved PFS (HR = 0.74; 95% CI: 0.67, 0.80; *P* < 0.001), and reduced recurrence rates (RR = 0.72; 95% CI: 0.60, 0.85; *P* < 0.001). Subgroup analysis showed that factors including area, histology, and source of the control group were significantly related to improved OS and PFS in patients with eEOC. Sensitivity analysis showed that the combined results were stable and reliable, and no significant publication bias was observed.

**Conclusions:**

Patients with eEOC can benefit from lymphadenectomy, with improved survival outcomes (OS and PFS) and a lower recurrence rate.

**Supplementary Information:**

The online version contains supplementary material available at 10.1186/s12905-023-02615-6.

## Highlights

1. The clinical outcomes of eEOC patients who did and did not undergo lymphadenectomy were compared.

2. Lymphadenectomy was associated with better survival outcomes in patients with eEOC.

3. Lymphadenectomy was associated with a lower recurrence rate in patients with eEOC.

**Supplementary Information:**

The online version contains supplementary material available at 10.1186/s12905-023-02615-6.

## Background

Epithelial ovarian cancer (EOC) is the leading cause of death among gynecological malignancies [1]. Due to the lack of early screening, ovarian cancer is usually diagnosed at an advanced stage [2]; however, approximately 30% of patients are diagnosed at an early stage (International Federation of Obstetrics and Gynecology, defined as FIGO stages I and II), and their 5-year survival rate is markedly higher than that of patients with advanced disease [3, 4].

In early-stage EOC (eEOC), surgical staging procedures, including systematic pelvic and para-aortic lymphadenectomies, are the basis for determining treatment options [5]. Lymph node dissection is helpful for accurately determining the disease stage and provides a reference for the subsequent formulation of adjuvant therapy [6]. However, the application of systematic lymphadenectomy remains controversial, particularly in its early stages. For example, Li et al. found that systematic pelvic and para-aortic lymphadenectomy did not significantly extend the overall survival of patients with eEOC [7]. Similar results were reported by Yoshihara et al. [8]. Conversely, Bizzarri and colleagues showed an improved 5-year disease-free survival after lymphadenectomy among eEOC [9]. Although several meta-analyses have also been designed to evaluate the clinical efficacy of lymphadenectomy in patients [10–14], these studies predominantly focused on advanced-stage EOC; therefore, whether lymphadenectomy provides a survival benefit in patients with eEOC remains unclear.

To determine the survival value of lymphadenectomy in patients with eEOC, we performed a meta-analysis to systematically evaluate the survival outcomes (including overall survival (OS) and progression-free survival (PFS) and recurrence rates of lymphadenectomy in patients with eEOC.

## Materials and methods

This meta-analysis was conducted in accordance with the Preferred Reporting Items for Systematic Reviews and Meta-Analyses (PRISMA) guidelines [15]. The PRISMA checklist is shown in Table S1.

### Selection strategy

Relevant studies were searched from the PubMed, Embase, Cochrane Library, and Web of Science databases without language restrictions. The search terms included the following key words: (“ovarian neoplasm” OR “ovarian cancer” OR “ovary neoplasm” OR “ovary cancer” OR “ovarian carcinoma” OR “ovary carcinoma”) AND (“lymphadenectomy” OR “lymph node dissection” OR “lymph node excision”) AND (“mortality” OR “survival” OR “death” OR “recurrence” OR “PFS” OR “OS”) up to May 16, 2022. The search strategy for each database is presented in Tables S2, S3, S4 and S5. To enroll more articles, the printout literature and reference lists of the included studies were manually searched.

### Inclusion and exclusion criteria

The inclusion criteria were as follows: (1) studies on patients diagnosed with eEOC confirmed by pathology, histology, or case records; (2) studies reporting on the survival or recurrence rate of lymphadenectomy vs. non-lymphadenectomy (NL) or comprehensive lymphadenectomy (CL, both pelvic and para-aortic lymphadenectomy) vs. pelvic lymphadenectomy/clinical lymph node evaluation (UCL); (3) prospective/retrospective cohort studies (PCS/RCS) or randomized controlled trials (RCTs); and (4) studies reporting hazard ratios (HR) with 95% confidence intervals (CIs) for PFS, OS, or number of recurrences.

The exclusion criteria were as follows: (1) non-research articles, such as reviews, comments, and conference summaries; (2) studies that only provided survival analysis results without reporting HRs (95% CI); and (3) duplicate studies or multiple articles reporting the same data; in this case, only the study with the most complete information was included.

### Data extraction and quality assessment

Two investigators independently screened the studies based on the above criteria. The following information was independently extracted by the two investigators: sample size, year of publication, name of the first author, publication year, research type, research area, recruitment time of participants, basic characteristics of the cases, histological subtype of ovarian cancer, staging, follow-up time, and clinical outcomes.

The Newcastle–Ottawa Scale (NOS) was used to assess the methodological quality of the included PCSs/RCSs from three perspectives: subject selection, comparability, and exposure [16]. In brief, studies with scores ranging from 7 to 9 points were considered high quality, 4–6 to points as moderate quality, and < 4 points as poor quality. Moreover, the quality of the included RCTs was assessed using the Cochrane Collaboration tool for assessing risk [17].

### Statistical analysis

All statistical analyses were performed using Stata software (version 12.0; Stata Corp., College Station, TX, USA). HRs with 95% CIs were used to evaluate the effect of lymphadenectomy on the prognostic survival (PFS and OS) of patients with eEOC. In addition, risk ratios (RRs) with 95% CIs were applied to assess the influence of lymphadenectomy on recurrence rates. Heterogeneity among studies was calculated using Cochran Q and *I*^*2*^ tests [18]. *P* < 0.05 and *I*^*2*^ > 50% suggested obvious heterogeneity among studies, and the random effects model was selected to calculate the pooled data; otherwise, a fixed effects model was selected (P ≥ 0.05, *I*^*2*^ ≤ 50%). To assess heterogeneity across studies, we conducted subgroup analyses based on the following factors: area (western vs. eastern), type of study (RCS vs. RCT), histology (various vs. specific), stage (≥ I vs. I only), source of the control group (NL vs. UCL), and use of multivariate analysis (yes vs. no).

Furthermore, Egger’s test and funnel charts were used to evaluate publication bias [19], and sensitivity analysis was performed to examine the stability of the results.

## Results

### Results of the study selection

**Fig. 1:**
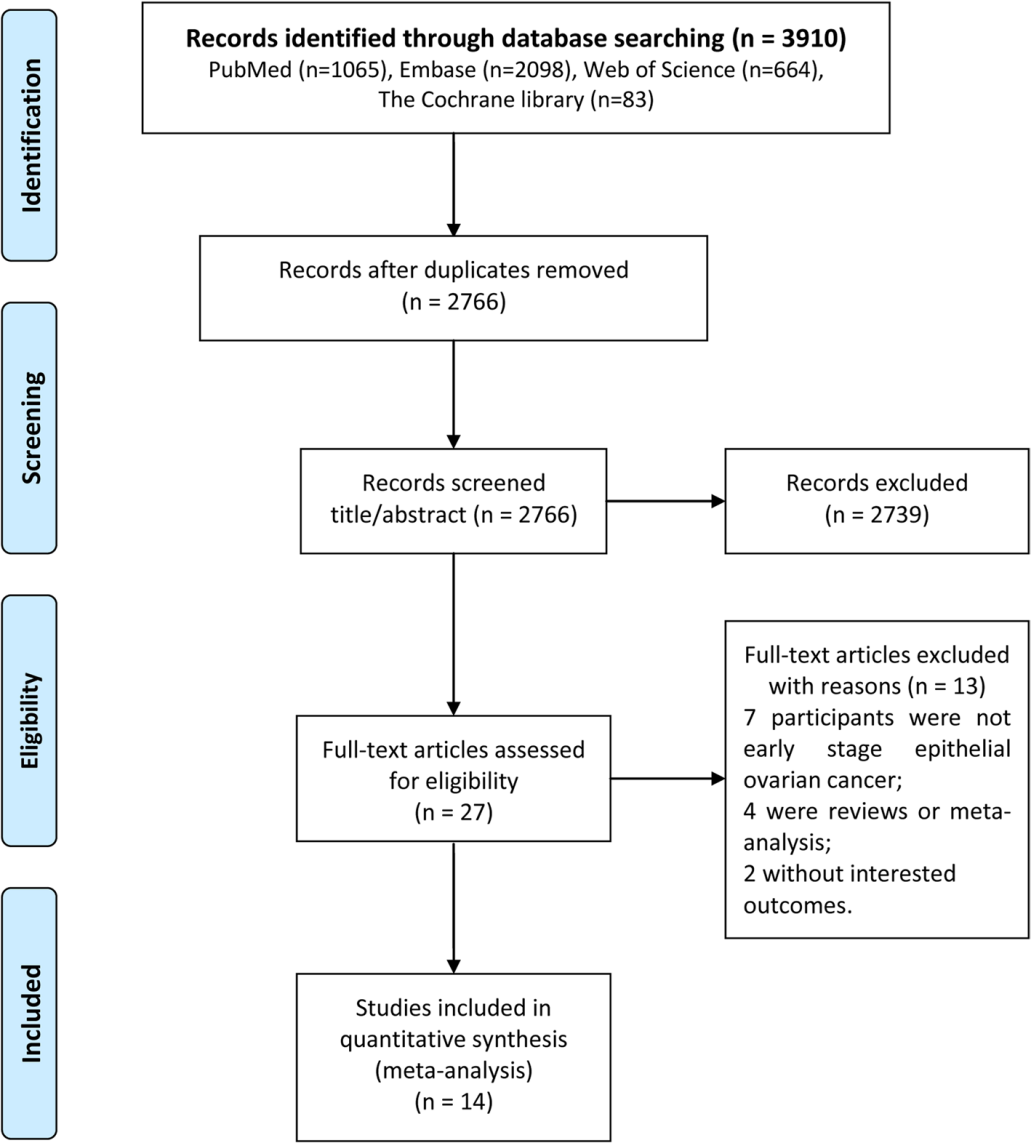
Detailed flow chart of the study selection process

The literature search process is illustrated in Fig. 1. In this study, 3910 studies were initially searched, including 1065, 2098, 83, and 664 articles from the PubMed, Embase, Cochrane Library, and Web of Science databases, respectively. After removing duplicate documents, 2766 articles remained. Following the analysis of titles and abstracts, 2739 articles were excluded from further consideration. The remaining 27 articles were fully reviewed, and 13 were excluded because they did not meet the inclusion criteria. A manual search of the references in these studies did not reveal any eligible studies. Finally, 14 articles were included in this meta-analysis [5, 8, 9, 20–30].

### Characteristics of the included studies and quality assessment

Detailed characteristics of each study are listed in Table 1. Among the included studies, 13 had an RCS design, while only one [24] was designed as an RCT. Two studies [9, 21] enrolled a small number of patients with stage III disease, whereas the remaining studies included patients with stage I or stage I–II disease. All studies were published between 2003 and 2021 and were conducted in various countries, including China, the United States, Japan, and Italy. The sample size of each study ranged from 62 to 13,117. Finally, 22,178 subjects were enrolled in this meta-analysis, including 12,167 cases in the lymphadenectomy group and 10,011 cases in the control (non-lymphadenectomy) group. Notably, there was no significant difference in age between the two groups in any of the studies, except for the study by Bizzarri et al. [9].

**Table 1:** Characteristics of the included studies

Study (Area)	Design	ACT, Yes/no	FIGO stage	Histology	Follow-up (months)	Group (n)	Number of resected LN	Age, years	Grade (%, 1/2/3/UN)	Stage (%, I/II/III)	Outcomes
Abe 2010 (Japan) [21]	RCS	57/0	I-III	Various	31 (2-83)	L (40)	9-80	55.5 (43, 72)	15/10/10/37	67.5/22.5/10	PFS
						NL (22)	0	45.1 (20, 63)	13.6/4.5/0/59	63.6/36.4/0	
Bizzarri 2021 (Italy, Germany) [9]	RCS	510/0	IA- IIIA1	Various	63 (5-342)	L (510)	32 (1-149)	54.6±10.8	4.1/22.7/72.5/0.6	61.4/27.3/11.4	Recurrence, PFS
		129/0				NL (129)	0	60±12.1 *	10.9/22.5/66.7/0	65.3/34.7/0	
Chen 2021 (USA, SEER) [22]	RCS	NR	I	Various	NR	L (581)	≥1	≤45	33.4/23.2/12.6/27.0	98.6/0/0	PFS
						NL (581)	0		35.6/22.9.5/28.6	97.2/0/0	
Deng 2021 (China) [5]	RCS	289/30	I-II	Various	69 (4-195)	L (319)	25 (1-64)	47 (17, 76)	17.9/24.8/53.9/3.4	66.1/33.9/0	Recurrence
		70/11				NL (81)	0	47 (20, 85)	34.6/25.9/25.9/13.6	67.9/32.1/0	
Ho 2003 (Taiwan) [23[	RCS	12/0	I	CCOC	36 (11-130)	L (12)	36 (5-49)	56.05±9.07	NR	100/0/0	Recurrence
		8/0				NL (8)	0	55.12±7.05			
Maggioni 2006 (Italy) [24]	RCT	91/47	I-II	Various	87.8 (62.7, 120.6)	CL (138)	47 (33, 63)	51 (43, 60)	21.7/21.0/52.2/5.1	73.9/23.9/0	Recurrence, PFS, OS
		73/57				UCL (130)	5.5 (0, 12)	52 (44, 59)	15.4/31.5/50.0/3.1	69.2/30.0/0	
Matsuo 2018 (USA, SEER) [20]	RCS	NR	I-II	Various	Median 85.2	CL (6349)	>12	56 (20)	21.0/26.3/30.6/22.1	73.0/27.0/0	PFS
						UCL (6768)	1-12				
Nasioudis 2019 (USA) [25]	RCS	1322/2920	I	MOC	Range 1-144	L (3367)	NR	51 (21)	38.5/32.5/8.7/21.3	100/0/0	OS
						NL (1444)	0				
Oshita 2013 (Japan) [26]	RCS	248/36	I-II	Various	64.9 (42.3, 90.8)	L (284)	34 (20-52)	53.5 (17-80)	NR	71.8/28.2/0	Recurrence, OS
		94/44				NL (138)	0	52 (16-91)	NR	84.9/15.1/0	
Svolgaard 2014 (Denmark) [27]	RCS	NR	I	Various	38 (1-76)	L (216)	NR	59 (13-90)	38.1/25.4/18.0/18.5	100/0/0	OS
						NL (411)	0				
Yamazaki 2018 (Japan) [28]	RCS	93/34	I-II	CCOC	1-72	CL (79)	59 median	53.9±10.1	NR	92.4/7.6/0	PFS, OS
						UCL (48)	NR	53.8±9.3	NR	81.3/18.7/0	
Yoshihara 2020 (Japan) [29]	RCS	31/24	I	MOC	NR	CL (55)	NR	50.7±11.4	NR	100/0/0	Recurrence, PFS, OS
		64/67				UCL (131)	NR	46.4±17.6			
Yoshihara 2021 (Japan) [8]	RCS	104/41	I	EOC	NR	CL (145)	NR	53.0±9.5	38.6/33.1/13.8/14.5	100/0/0	Recurrence, OS
		69/45				UCL (114)	NR	53.4±14.6	44.7/21.9/4.4/28.9	100/0/0	
Zhao 2017 (China) [30]	RCS	Aug-70	I	EOC	74.5 (56, 117)	L (72)	18 (2-48)	48.37±13.29	44.9/33.3/21.8/0	100/0/0	Recurrence, PFS
						NL (6)	0				

*ACT *Adjuvant chemotherapy, *CCOC *Clear cell ovarian cancer, *EOC *Endometrioid ovarian cancer, *MOC *Mucinous ovarian cancer, *OS *Overall survival, *PFS *Progression-free survival, *USA *the United States of America, *L *pelvic and para-aortic lymphadenectomy or pelvic lymphadenectomy, *CL *Comprehensive lymphadenectomy, both pelvic and para-aortic lymphadenectomy, *UCL *Pelvic lymphadenectomy or clinical lymph node evaluation, *NL *No lymphadenectomy, *RCS *Retrospective cohort study, *RCT *Randomized controlled trial, *SEER *Surveillance, Epidemiology, and End Results, *NR *Not reported, *UN *Unknown; *LN* lymph node. *, *P* < 0.05

The quality of included studies was also assessed. Briefly, the NOS scores of the 13 RCS ranged from 5 to 8 points, suggesting that the methodological quality of the included RCS was moderate. For the RCT, the main biases were related to implementation and measurement. Overall, the degree of bias was moderate.

### Meta-analysis of clinical prognosis and recurrence rate

**Fig. 2:**
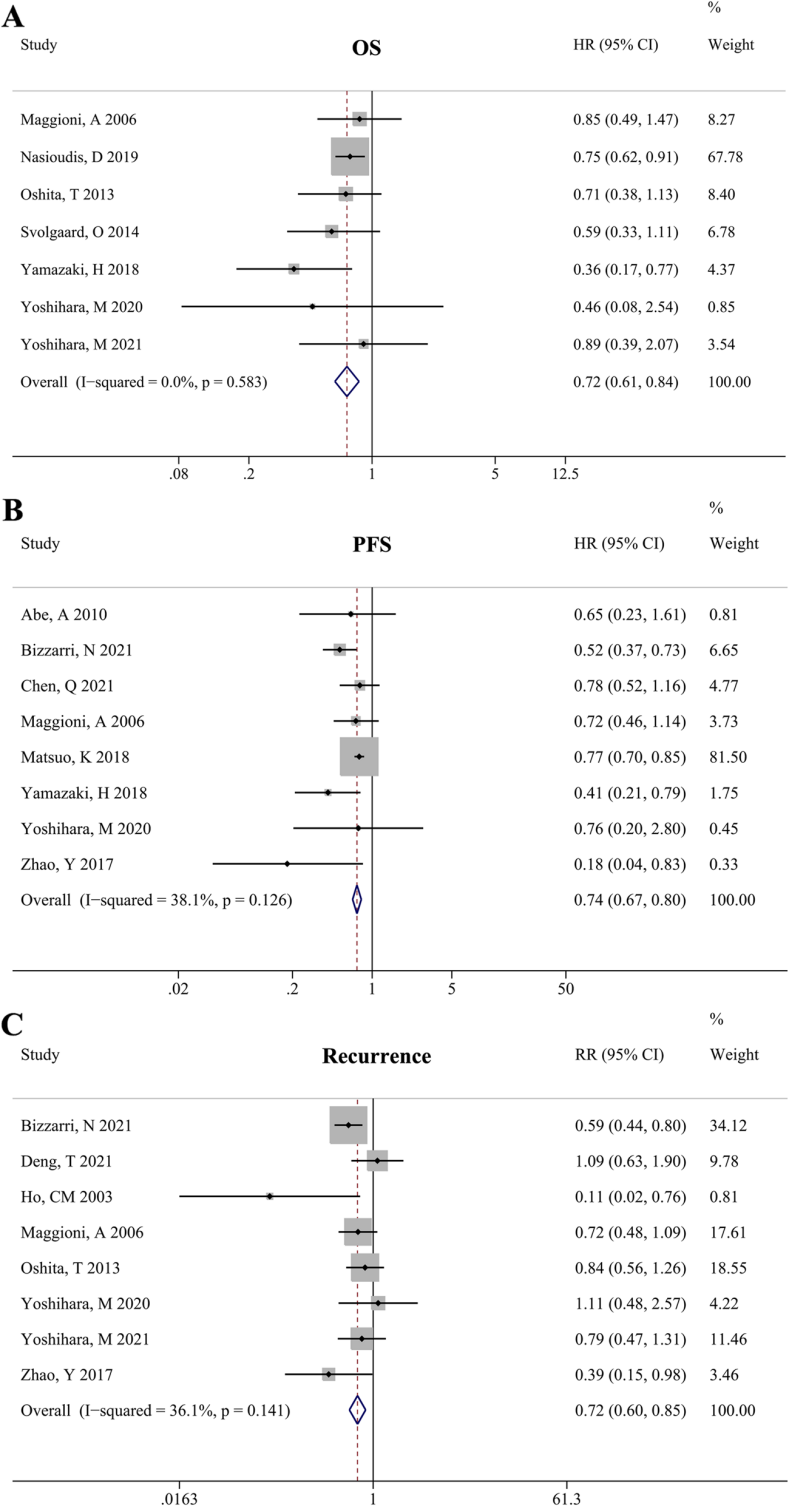
Forest plot for survival outcomes and recurrence in early-stage epithelial ovarian cancer patients undergoing lymphadenectomy. **A** overall survival; **B**: progression-free survival; **C**: recurrence rate

Seven studies reported OS outcomes. No significant heterogeneity was observed among the studies (*I*^*2*^ = 0%, *P* = 0.583); therefore, a fixed-effects model was used for the meta-analysis. The results showed that lymphadenectomy was associated with a better OS in patients with eEOC (HR = 0.72, 95% CI: 0.61–0.84, *P* < 0.001, Fig. 2A).

Eight studies recorded the PFS data of patients. No significant heterogeneity was observed among the studies (*I*^*2*^ = 38.1%, *P* = 0.126); thus, a fixed-effects model was used to calculate the pooled results. The results showed that lymphadenectomy was associated with improved PFS in patients with eEOC (HR = 0.74, 95% CI: 0.67–0.80, *P* < 0.001, Fig. 2B). In addition, eight articles reported recurrence data, and no significant heterogeneity was found (*I*^*2*^ = 36.1%, *P* = 0.141). Overall, the pooled results showed a significant difference in recurrence between the lymphadenectomy and control groups (RR = 0.72, 95% CI: 0.60–0.85, *P* < 0.001, Fig. 2C), indicating that lymphadenectomy could decrease the recurrence rates of patients with eEOC.

### Subgroup analysis of OS

The results of the subgroup analyses are presented in Table 2. No significant difference in OS was observed between the lymphadenectomy and control groups in the univariate analysis (*P* = 0.796) or the RCT (*P* = 0.562) subgroups. In addition, subgroup analysis stratified by area (OR = 0.74, 95% CI: 0.62–0.89, *P* = 0.001 for western areas; OR = 0.61, 95% CI: 0.42–0.90, *P* = 0.012 for eastern areas), histology (OR = 0.72, 95% CI: 0.52–0.99, *P* = 0.0146 for various; OR = 0.72, 95% CI: 0.60–0.86, *P* < 0.001 for specific), stage (OR = 0.66, 95% CI: 0.47–0.93, *P* = 0.019 for stage ≥ I; OR = 0.74, 95% CI: 0.62–0.88, *P* = 0.001 for stage I only), and source of the control group (OR = 0.73, 95% CI: 0.61–0.87, *P* < 0.001 for NL; OR = 0.67, 95% CI: 0.46–0.98, *P* = 0.039 for UCL) showed that lymphadenectomy was significantly associated with better OS.

**Table 2:** Outcomes of the subgroup analysis

Subgroup	No. of studies	Heterogeneity test		Effect size	
		I^2^ (%)	P_H_	OR (95% CI)	*P*-value
**OS**	7	0	0.583	0.72 (0.61, 0.84)	< 0.001
Area					
Western	3	0	0.672	0.74 (0.63, 0.89)	0.001
Eastern	4	2.5	0.38	0.61 (0.42, 0.90)	0.012
Type of study					
RCS	6	0	0.504	0.71 (0.60, 0.84)	< 0.001
RCT	1	NA	NA	0.85 (0.49, 1.47)	0.562
Histology					
Various	3	0	0.682	0.72 (0.52, 0.99)	0.046
Specific^a^	4	23.7	0.267	0.72 (0.60, 0.86)	< 0.001
MOC	2	0	0.576	0.75 (0.62, 0.90)	0.003
CCOC	1	NA	NA	0.36 (0.17, 0.77)	0.008
EOC	1	NA	NA	0.89 (0.39, 2.07)	0.796
Stage					
≥ I	3	40.4	0.187	0.66 (0.47, 0.93)	0.019
I only	4	0	0.79	0.74 (0.62, 0.88)	0.001
Control group					
NL	3	0	0.756	0.73 (0.61, 0.87)	< 0.001
UCL	4	24.3	0.265	0.67 (0.46, 0.98)	0.039
Multivariate analysis					
Yes	6	0	0.489	0.71 (0.61, 0.84)	< 0.001
No	1	NA	NA	0.89 (0.39, 2.07)	0.796
**PFS**	8	38.1	0.126	0.74 (0.67, 0.80)	< 0.001
Area					
Western	4	37.6	0.186	0.75 (0.68, 0.82)	< 0.001
Eastern	4	0	0.453	0.46 (0.28, 0.74)	0.001
Type of study					
RCS	7	46.9	0.079	0.74 (0.67, 0.81)	< 0.001
RCT	1	NA	NA	0.72 (0.46, 1.13)	0.156
Histology					
Various	5	18.2	0.299	0.75 (0.68, 0.82)	< 0.001
Specific^a^	3	0	0.373	0.41 (0.24, 0.71)	0.002
MOC	1	NA	NA	0.76 (0.20, 2.80)	0.676
CCOC	1	NA	NA	0.41 (0.21, 0.80)	0.008
EOC	1	NA	NA	0.18 (0.04, 0.82)	0.027
Stage					
≥ I	5	49.5	0.095	0.74 (0.67, 0.81)	< 0.001
I only	3	40.6	0.186	0.71 (0.49, 1.03)	0.074
Control group					
NL	4	37.1	0.189	0.60 (0.47, 0.77)	< 0.001
UCL	4	13.2	0.327	0.76 (0.69, 0.83)	< 0.001
**Recurrence**	8	36.1	0.141	0.72 (0.60, 0.85)	< 0.001
Area					
Western	2	0	0.434	0.63 (0.50, 0.81)	< 0.001
Eastern	6	39.4	0.143	0.82 (0.64, 1.05)	0.114
Type of study					
RCS	7	45.2	0.09	0.72 (0.59, 0.87)	0.001
RCT	1	NA	NA	0.72 (0.48, 1.09)	0.125
Histology					
Various	4	33	0.215	0.75 (0.58, 0.95)	0.018
Specific^a^	4	53.4	0.092	0.60 (0.31, 1.16)	0.13
MOC	1	NA	NA	1.11 (0.48, 2.57)	0.805
CCOC	1	NA	NA	0.11 (0.02, 0.76)	0.025
EOC	2	41.4	0.193	0.62 (0.32, 1.19)	0.149
Stage					
≥ I	4	33	0.215	0.75 (0.58, 0.95)	0.018
I only	4	53.4	0.092	0.60 (0.31, 1.16)	0.13
Control group					
NL	5	58.3	0.048	0.67 (0.45, 0.99)	0.046
UCL	3	0	0.669	0.79 (0.58, 1.06)	0.116

^a^Specific: Only one subtype was included, such as mucinous ovarian cancer (MOC), endometrioid ovarian cancer (EOC), or clear cell ovarian cancer (CCOC)

### Subgroup analysis of PFS

As HR values could only be obtained after multifactor correction for PFS, subgroup analysis based on multivariate analysis was not conducted. Nonetheless, the results showed that statistical significances were observed in the subgroup stratified by area (OR = 0.75, 95% CI: 0.68–0.82, *P* < 0.001 for western; OR = 0.46, 95% CI: 0.28–0.74, *P* = 0.001 for eastern), histology (OR = 0.75, 95% CI: 0.68–0.82, *P* < 0.001 for various; OR = 0.41, 95% CI: 0.24–0.71, *P* = 0.002 for specific), and source of the control group (OR = 0.60, 95% CI: 0.47–0.77, *P* < 0.001 for NL; OR = 0.76, 95% CI: 0.69–0.83, *P* < 0.001 for UCL), indicating that these factors affected the PFS of patients with eEOC. However, RCT (*P* = 0.156) subgroup and patients with stage I disease (*P* = 0.074) did not show a significant impact on PFS.

### Subgroup analysis of recurrence

Recurrence outcomes were compared based on univariate analysis. The results were consistent among subgroups stratified by western area (OR = 0.63, 95% CI: 0.50–0.81, *P* < 0.001), RCS study (OR = 0.72, 95% CI: 0.59–0.87, *P* = 0.001), “various” histology (OR = 0.75, 95% CI: 0.58–0.95, *P* = 0.018), stage ≥ I (OR = 0.75, 95% CI: 0.58–0.95, *P* = 0.018), and NL as the source of the control group (OR = 0.67, 95% CI: 0.45–0.99, *P* = 0.046). However, the association between eEOC and recurrence was not significant when stratified by eastern area (*P* = 0.114), RCT (*P* = 0.125), “specific” histology (*P* = 0.130), stage I (*P* = 0.130), or UCL as the source of the control group (*P* = 0.116).

### Sensitivity analysis and publication bias

**Fig. 3:**
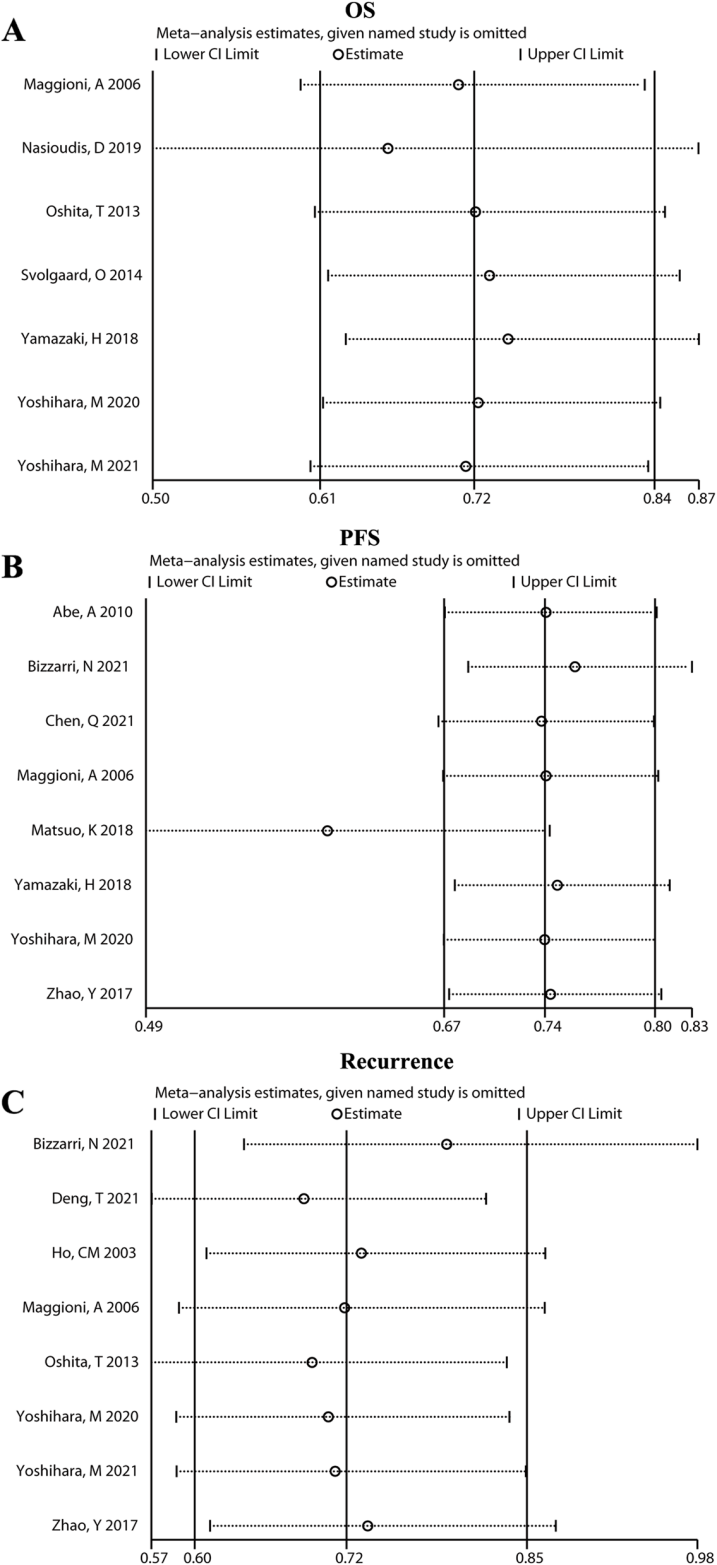
Sensitivity analysis of survival outcomes and recurrence in early-stage epithelial ovarian cancer patients undergoing lymphadenectomy. **A** overall survival; **B**: progression-free survival; **C**: recurrence rate

Sensitivity analysis was performed to assess the stability of the results. As shown in Fig. 3A, the finally results for OS, PFS, and recurrence rates did not change after excluding the studies one a time (Fig. 3A C), indicating the stability of our findings.

### Publication bias

**Fig. 4:**
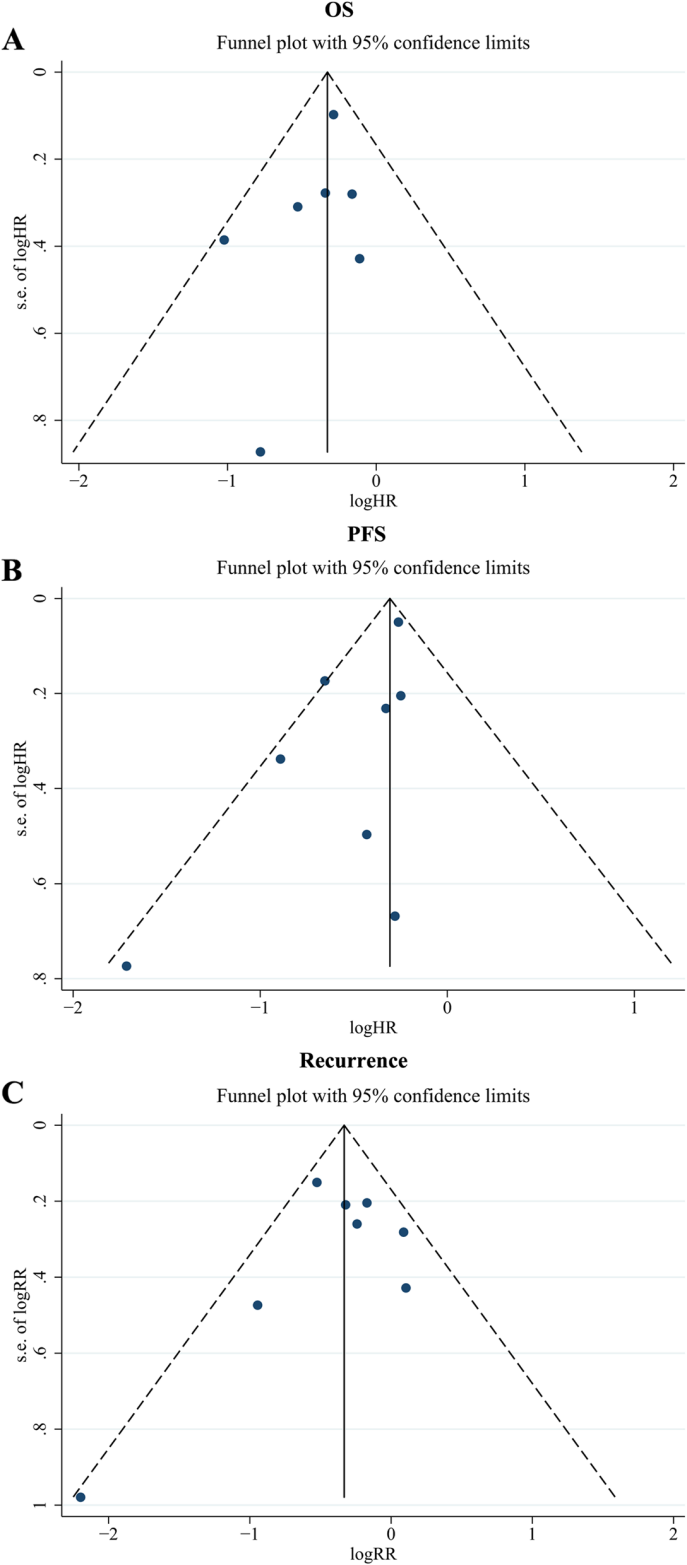
Funnel plot for the detection of publication bias. **A**: overall survival; **B**: progression-free survival; **C**: recurrence rate

Egger test results for OS, PFS, and recurrence rates were 0.313, 0.071, and 0.643, respectively. In addition, the symmetrical funnel chart showed that the scattered-point distribution had good symmetry, suggesting that no significant bias was observed among the studies for the three outcomes (Fig. 4A C).

## Discussion

eEOC is one of the most important health problems worldwide, and lymphatic metastasis is an accepted predictor of survival outcomes. Systematic lymphadenectomy is a major surgical procedure for eEOC; however, the clinical efficacy of lymphadenectomy remains controversial [24, 28]. Therefore, we performed a meta-analysis to systematically investigate the clinical outcomes of lymphadenectomy in the treatment of eEOC. Overall, 14 studies (13 RCS and one RCT) were included. Pooled analysis revealed that lymphadenectomy was associated with improved OS and PFS as well as a lower recurrence rate. Furthermore, the subgroup analysis showed that the area, histology, stage, and source of the control group may have influenced the effect of lymphadenectomy on OS improvement. In addition, in the control group, factors such as area, histology, and source were significantly associated with improved PFS in patients with eEOC. Overall, we conclude that patients with eEOC may benefit from lymphadenectomies.

In eEOC, the risk of occult pelvic and/or para-aortic lymph node metastasis has been reported to be 6.1–29.6%, with an average incidence of 14.2% [31]. Ovarian cancer is a heterogeneous disease with diverse oncology properties [32]. Among different histological types, the serous subtype had the highest incidence of lymph node metastasis (23.3%), whereas the mucinous subtype had the lowest incidence (2.6%) [31]. Hence, lymphadenectomy should be avoided in patients with mucinous ovarian and low-grade serous cancer [33].

In this meta-analysis, we observed that lymphadenectomy was associated with improved OS and PFS in patients with clear cell ovarian cancer, which was also observed by Yamazaki et al. [28]. However, the number of mucinous ovarian cancer studies was very small (only to 1–2 articles), preventing us from drawing conclusive results. Notably, the prognostic value of lymphadenectomy is supported by previous studies. Compared with inadequate dissection, Matsuo et al. demonstrated a 15–25% reduction in mortality with adequate lymphadenectomy [20]. Meanwhile, the number of removed lymph nodes positively correlated with the OS of patients with EOC at stages I–IIA and IIIA1 [34]. In addition, a previous study reported that the risk of recurrence in patients who underwent lymphadenectomy decreased from 30 to 22% [35]. These studies further support the findings of the present meta-analysis.

Although systematic lymphadenectomy in early-stage ovarian cancer allows clinicians to detect macroscopic nodal disease and identify patients who will benefit from adjuvant therapy, it is also a procedure with a considerable treatment burden that may be associated with intraoperative and postoperative complications [36]. Recently, a large-scale randomized clinical trial showed that systematic lymphadenectomy did not provide a survival benefit in patients with advanced ovarian cancer and was associated with a higher rate of postoperative complications, suggesting that these patients should not undergo lymphadenectomy [37]. Compared to patients with advanced disease, those with early disease typically do not present obvious symptoms and do not have massive ascites [38]. Furthermore, the prognosis for early-stage disease differs significantly, with a 10-year survival exceeding 80%, while the 5-year survival rate for advanced disease is only 30–40% [39]. Thus, the primary purpose of lymphadenectomy in early-stage disease is to stage and guide subsequent therapy, whereas in advanced disease, it is to achieve optimal tumor reduction. This may partly explain the differential effects of lymphadenectomy on early- and advanced-stage ovarian cancer treatment outcomes.

Another important consideration is the role of adjuvant chemotherapy, which is thought to improve the prognosis of EOC [40]. Platinum-based adjuvant chemotherapy improves survival and delays recurrence in patients with eEOC [41]. In addition, there is evidence that lymphadenectomy can remove occult microscopic lymphatic metastases; however, its effect is small compared to that of adjuvant platinum-based chemotherapy [5]. In contrast, Imterat et al. [33] revealed that adjuvant chemotherapy with carboplatin monotherapy after complete surgical staging is the standard of care for eEOC, indicating that systematic lymphadenectomy remains appropriate for patients with significant early-stage disease after careful evaluation of the histological subtypes. Additionally, apart from disease characteristics such as stage or histology, the decision to perform lymphadenectomy should consider the patient’s performance status. Di Donato et al. [42] found that in patients with advanced ovarian cancer accompanied by hepatobiliary involvement, highly complex multiorgan surgery, including lymphadenectomy, can provide a survival benefit. They also suggested that in actual clinical practice, the choice of surgery needs to be balanced between the expected improvement in survival and surgical morbidity or mortality. Hence, the implementation of systematic lymphadenectomy requires the consideration of the clinical presentation of each patient. Multicenter prospective studies are required to confirm our results.

These results indicate that lymphadenectomy is a relatively safe and acceptable procedure for eEOC, with a positive impact on overall survival. However, in the included studies, variations in the extent of lymphadenectomy were observed; for example, some clinicians performed only pelvic lymphadenectomy. In addition, the quality of systematic lymphadenectomy may vary slightly depending on the physician performing the procedure, and the number of removed lymph nodes is not necessarily indicative of the quality of the procedure. In this context, the potential role of sentinel lymph node (SLN) biopsy is of interest. SLN biopsy improves the detection of positive lymph nodes from a qualitative standpoint rather than solely relying on the number of lymph nodes dissected. SLN biopsy has been widely utilized in early cervical and endometrial cancers [43, 44], and recent clinical trials have indicated that SLN biopsies are feasible and reliable for early-stage ovarian cancer [45]. However, due to the complexity of ovarian cancer, technical challenges remain. Therefore, SLN technology for the treatment of early-stage ovarian cancer is still in the experimental stage.

This study has several strengths. Firstly, the studies were collected from multiple databases and included large sample sizes, confirming the accuracy and credibility of the present conclusions. Secondly, no obvious heterogeneity was observed among the included studies, indicating a high level of confidence in the pooled results. Thirdly, no significant publication bias was observed, and the sensitivity analysis confirmed the stability of the meta-analysis findings. Nevertheless, this meta-analysis had some limitations. Firstly, the therapeutic schedules, including surgical intervention and adjuvant treatment, were not consistent among the included studies. However, multivariate analysis adjusted for these factors may have affected the survival benefit of lymphadenectomy in patients with eEOC. Secondly, two of the 14 included studies had a large sample size [20, 25], which may have introduced sampling bias. Thirdly, most of the included studies were retrospective, and although multivariate analysis was performed in most cases, inconsistencies in the correction factors may also have affected the accuracy of the results. Lastly, the number of included studies was small in some subgroups, the definition of early-stage patients was not rigorously standardized, and two articles included a small number of stage III patients. Therefore, it is recommended that future studies employ more rigorous literature screening and high-quality designs with larger sample sizes to verify the conclusions of this study.

## Conclusion

In summary, the results of this study showed that lymphadenectomy may improve OS and PFS while reducing the risk of recurrence in patients with eEOC.

### Supplementary Information

**Additional file 1.**

## Data Availability

Data supporting the findings of this study are available in this article and the supplementary materials.

## Abbreviations

CL: Comprehensive lymphadenectomy
CI: Confidence interval
eEOC: Early-stage epithelial ovarian cancer
EOC: Epithelial ovarian cancer
HR: Hazard risk
NOS: Newcastle-Ottawa Scale
OS: Overall survival
PCS: Prospective cohort study
PFS: Progression-free survival
RCS: Retrospective cohort study
RCT: Randomized controlled trial
RR: Risk ratio
UCL: Pelvic lymphadenectomy/clinical lymph node evaluation
SLN: Sentinel lymph node
