# Supplementary material for: Influence of lymphadenectomy on survival and recurrence in patients with early-stage epithelial ovarian cancer: a meta-analysis
Source: BMC Womens Health. 2023 Sep 4;23:474. doi: 10.1186/s12905-023-02615-6 (PMC10478186; doi:10.1186/s12905-023-02615-6)
Supplement: Supplementary file 1 — Additional file 1. [file 12905_2023_2615_MOESM1_ESM.zip › Supplementary Tables/Supplementary Table 2-5.docx]

**Table S2.** **Search strategy and results of PubMed (retrieval time: 20220516)**

| Search | Query | Items found |
| --- | --- | --- |
| #1 | ("lymph node dissection"[All Fields] OR "lymph node excision"[MeSH Terms] OR "lymph node excision"[All Fields] OR "lymphadenectomies"[All Fields] OR "lymphadenectomy"[All Fields]) | 69980 |
| #2 | ("ovarian neoplasms"[MeSH Terms] OR "ovarian neoplasms"[All Fields] OR "ovarian cancer"[All Fields] OR "ovarian carcinoma"[All Fields] OR "ovary carcinoma"[All Fields] OR "ovary cancer"[All Fields] OR "ovary neoplasm"[All Fields]) | 112500 |
| #3 | "mortality"[MeSH Terms] OR "mortality"[All Fields] OR "mortalities"[All Fields] OR "mortality"[MeSH Subheading] OR "death"[MeSH Terms] OR "death"[All Fields] OR "deaths"[All Fields] OR "survival"[MeSH Terms] OR "survival"[All Fields] OR "survivability"[All Fields] OR "survivable"[All Fields] OR "survivals"[All Fields] OR "survive"[All Fields] OR "survived"[All Fields] OR "survives"[All Fields] OR "surviving"[All Fields] OR "OS"[All Fields] OR "PFS"[All Fields] OR "RFS"[All Fields] OR "recurrance"[All Fields] OR "recurrence"[MeSH Terms] OR "recurrence"[All Fields] OR "recurrences"[All Fields] OR "recurrencies"[All Fields] OR "recurrency"[All Fields] OR "recurrent"[All Fields] OR "recurrently"[All Fields] OR "recurrents"[All Fields] OR "relapse"[All Fields] OR "relapses"[All Fields] OR "relapsing"[All Fields] OR "relapsed"[All Fields] OR "relapser"[All Fields] OR "relapsers"[All Fields] | 3802271 |
| #4 | #1 AND #2 AND #3 | 1065 |

**Table S3. Search strategy and results of Embase (retrieval time: 20220516)**

| Search | Query | Items found |
| --- | --- | --- |
| #1 | ('lymph node dissection'/exp OR 'lymph node dissection' OR 'lymphadenectomy'/exp OR lymphadenectomy OR 'lymph node excision'/exp OR 'lymph node excision') | 87654 |
| #2 | ('ovarian neoplasms'/exp OR 'ovarian neoplasms' OR 'ovarian cancer'/exp OR 'ovarian cancer' OR 'ovarian carcinoma'/exp OR 'ovarian carcinoma' OR 'ovary carcinoma'/exp OR 'ovary carcinoma' OR 'ovary cancer'/exp OR 'ovary cancer' OR 'ovary neoplasm'/exp OR 'ovary neoplasm') | 158113 |
| #3 | ('mortality'/exp OR mortality:ab,ti OR 'death'/exp OR death:ab,ti OR 'survival'/exp OR survival:ab,ti OR os:ab,ti OR pfs:ab,ti OR rfs:ab,ti OR 'recurrence'/exp OR recurrence:ab,ti OR 'relapse'/exp OR relapse:ab,ti) | 4160473 |
| #4 | #1 AND #2 AND #3 | 2098 |

**Table S4. Search strategy and results of Web of Science (retrieval time: 20220516)**

| Search | Query | Items found |
| --- | --- | --- |
| #1 | ALL FIELDS: (("lymph node dissection") OR (Lymphadenectomy) OR (Lymph Node Excision)) | 43449 |
| #2 | ALL FIELDS: ("ovarian neoplasms" OR "ovarian cancer" OR "ovarian carcinoma" OR "ovary carcinoma" OR "ovary cancer" OR "ovary neoplasm") | 98855 |
| #3 | ALL FIELDS: (mortality OR death OR survival OR OS OR PFS OR RFS OR recurrence OR relapse) | 3288556 |
| #4 | #1 AND #2 AND #3 | 664 |

**Table S5. Search strategy and results of the Cochrane library (retrieval time: 20220516)**

| Search | Query | Items found |
| --- | --- | --- |
| #1 | MeSH descriptor: [Lymph Node Excision] explode all trees | 1442 |
| #2 | ("lymph node dissection" OR "lymph node excision" OR "lymphadenectomies" OR "lymphadenectomy"):ti,ab,kw (Word variations have been searched) | 4612 |
| #3 | #1 OR #2 | 4853 |
| #4 | MeSH descriptor: [Ovarian Neoplasms] explode all trees | 2177 |
| #5 | ("ovarian neoplasms" OR "ovarian cancer" OR "ovarian carcinoma" OR "ovary carcinoma" OR "ovary cancer" OR "ovary neoplasm"):ti,ab,kw (Word variations have been searched) | 7137 |
| #6 | #4 OR #5 | 7180 |
| #7 | MeSH descriptor: [Mortality] explode all trees | 14068 |
| #8 | MeSH descriptor: [Death] explode all trees | 2348 |
| #9 | MeSH descriptor: [Survival] explode all trees | 134 |
| #10 | (mortality OR death OR surviv* OR OS OR PFS OR RFS OR recurren* OR relapse):ti,ab,kw (Word variations have been searched) | 304212 |
| #11 | #7 OR #8 OR #9 OR #10 | 304721 |
| #12 | #3 AND #6 AND #11 | 86 |
| #13 | #12 in Trials | 83 |
